# Supplementary material for: Global effects of land-use intensity on local pollinator biodiversity
Source: Nat Commun. 2021 May 18;12:2902. doi: 10.1038/s41467-021-23228-3 (PMC8131357; doi:10.1038/s41467-021-23228-3)
Supplement: Supplementary file 1 — Supplementary Information [file 41467_2021_23228_MOESM1_ESM.pdf]

Global effects of land-use intensity on local pollinator biodiversity

Supplementary information

Contents

Supplementary Figures ... 3
Supplementary Figure 1 ... 3
Supplementary Figure 2 ... 4
Supplementary Figure 3 ... 5
Supplementary Figure 4 ... 6
Supplementary Figure 5 ... 7
Supplementary Figure 6 ... 8
Supplementary Figure 7 ... 9
Supplementary Figure 8 ... 10
Supplementary Figure 9 ... 11
Supplementary Figure 10 ... 12
Supplementary Figure 11 ... 13
Supplementary Figure 12 ... 14
Supplementary Figure 13 ... 15
Supplementary Figure 14 ... 16
Supplementary Tables ... 17
Supplementary Table 1 ... 17
Supplementary Table 2 ... 17
Supplementary Table 3 ... 18
Supplementary Table 4 ... 19
Supplementary Table 5 ... 21
Supplementary Table 6 ... 22
Supplementary Table 7 ... 23
Supplementary Table 8 ... 23
Supplementary Table 9 ... 24
Supplementary Table 10 ... 24
Supplementary Table 11 ... 25
Supplementary Table 12 ... 26
Supplementary Table 13 ... 27
Supplementary Table 14 ... 28

Supplementary Table 15 29
Supplementary Table 16 29
Supplementary Table 17 30
Supplementary Table 18 31
Supplementary Table 19 32
Supplementary Table 20 33

Supplementary Figures

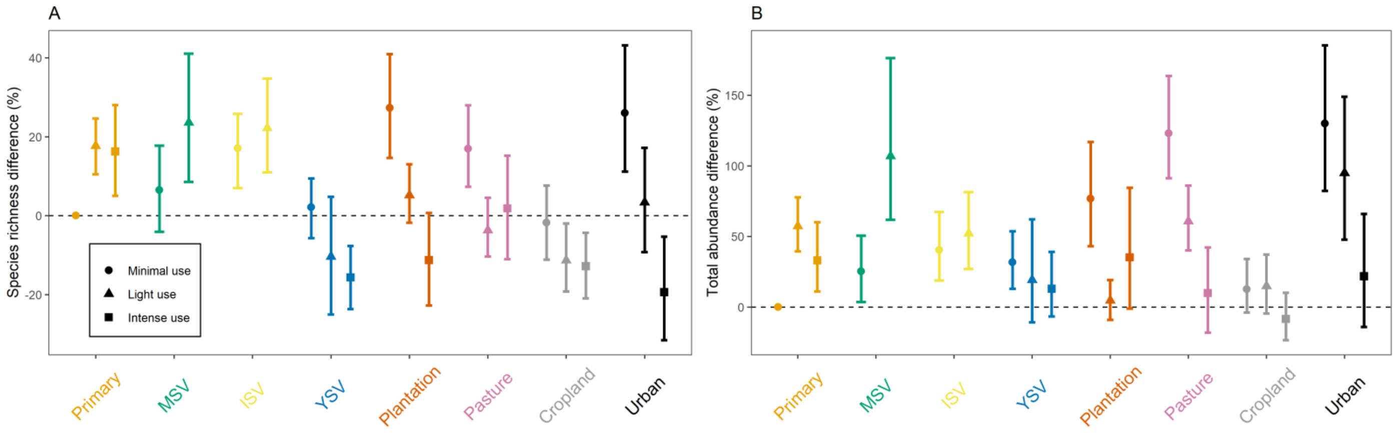

Supplementary Figure 1. Response of pollinator biodiversity to land-use type and land-use intensity for species richness and total abundance, both fit using generalised linear mixed-effects models. A) species richness, fit using a poisson error generalised linear mixed-effects model; and B) total abundance, fit using a zero-inflated negative binomial error generalised linear mixed-effects model. Colours represent land-use type: orange (primary vegetation, Primary), green (mature secondary vegetation, MSV), yellow (intermediate secondary vegetation, ISV), blue (young secondary vegetation, YSV), dark orange (plantation forest, Plantation), pink (pasture), grey (cropland), and black (urban), and point shape represents land-use intensity: circle (minimal uses), triangles (light use), and squares (intense use). Effect sizes were adjusted to a percentage by drawing fixed effects 1,000 times based on the variance-covariance matrix, expressing each fixed effect as a percentage of the baseline (primary vegetation minimal use), and then calculating the median value (shown as points), and 2.5th and 97.5th percentiles (shown as error bars).

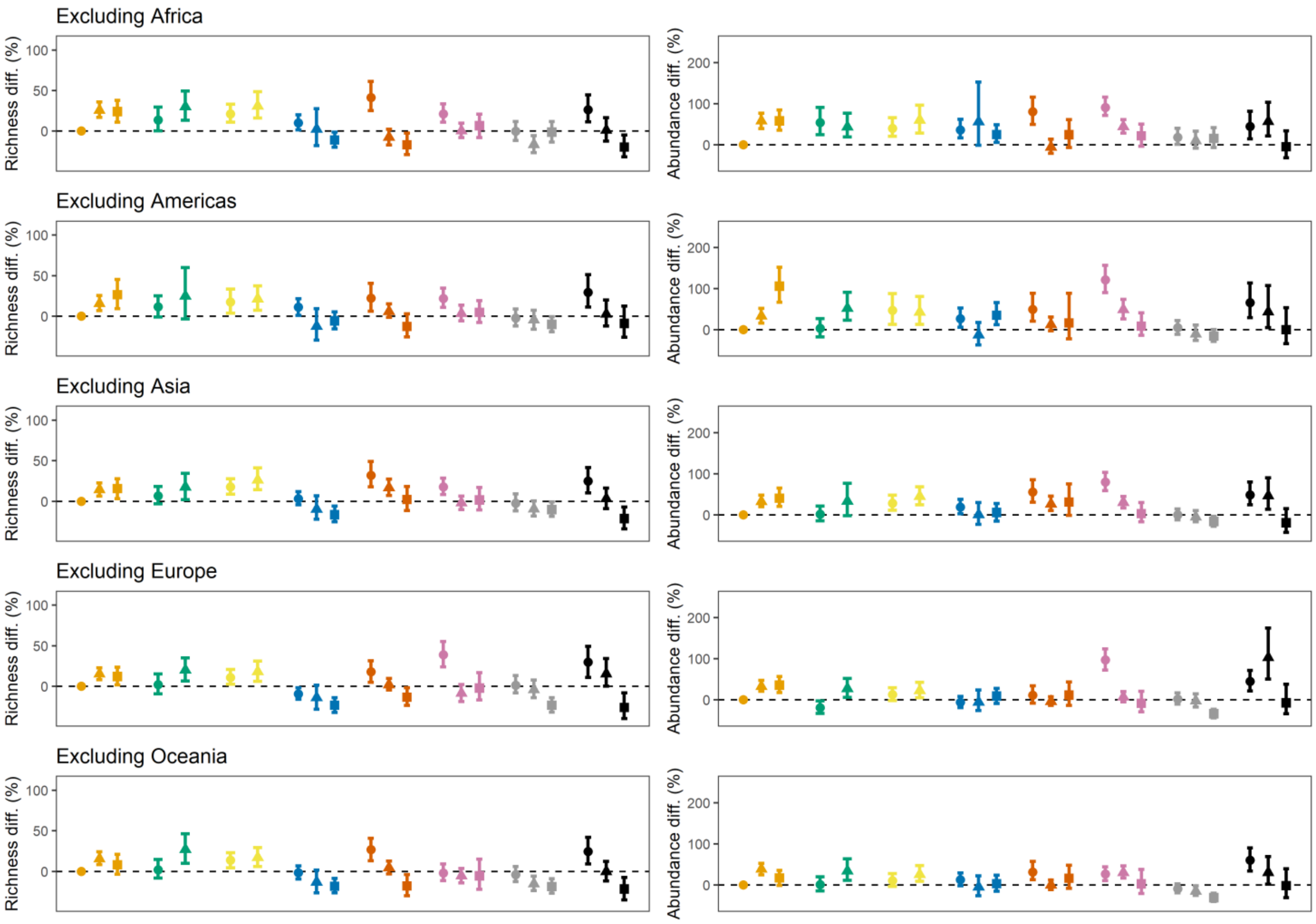

Supplementary Figure 2. Response of pollinator biodiversity to land-use type and land-use intensity for species richness and total abundance (fit using the approach in the main text), jack-knifed by UN regions (Africa, Americas, Asia, Europe, and Oceania). Colours represent land-use type: orange (primary vegetation), green (mature secondary vegetation), yellow (intermediate secondary vegetation), blue (young secondary vegetation), dark orange (plantation forest), pink (pasture), grey (cropland), and black (urban), and point shape represents land-use intensity: circle (minimal uses), triangles (light use), and squares (intense use). Effect sizes were adjusted to a percentage by drawing fixed effects 1,000 times based on the variance-covariance matrix, expressing each fixed effect as a percentage of the baseline (primary vegetation minimal use), and then calculating the median value (shown as points), and 2.5th and 97.5th percentiles (shown as error bars).

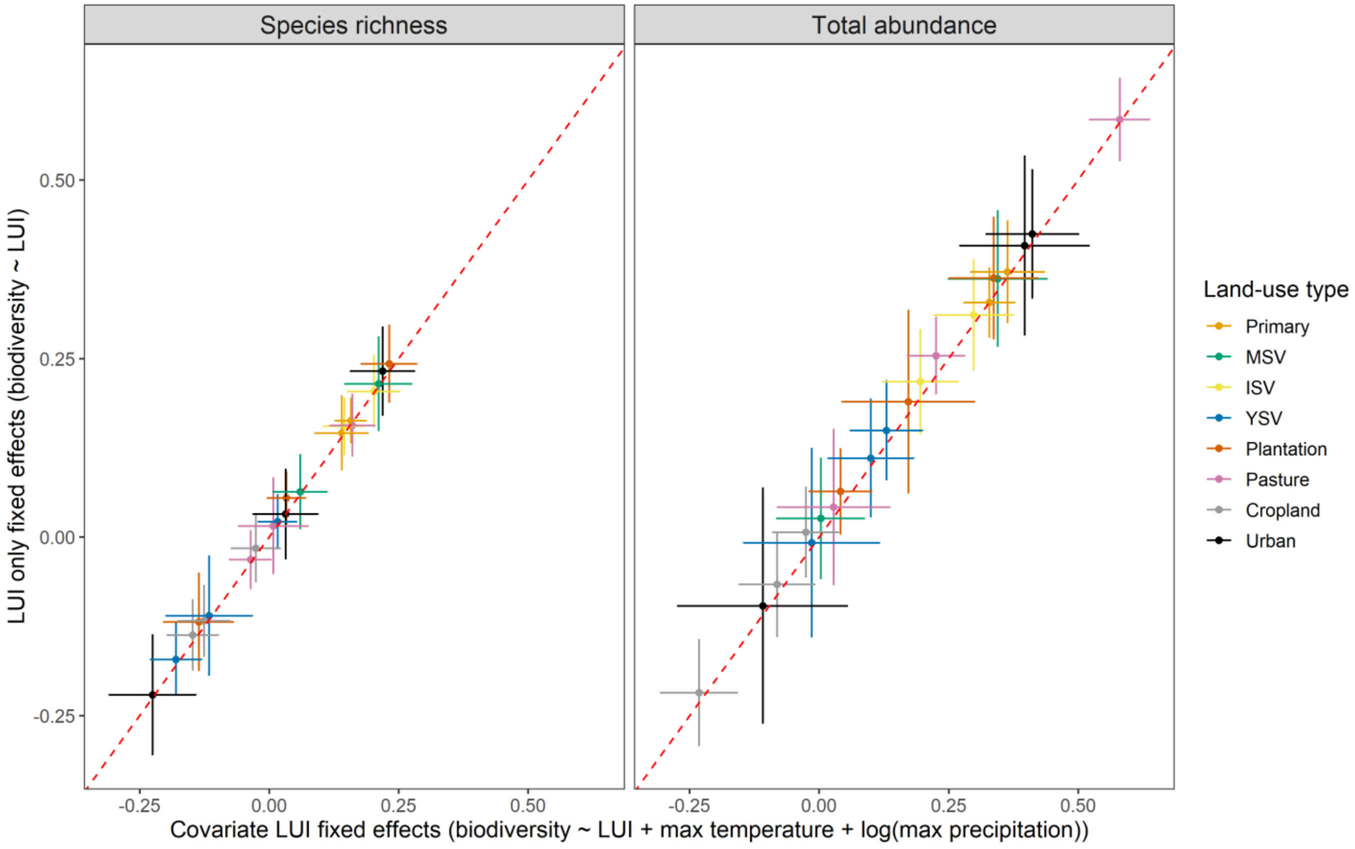

Supplementary Figure 3. Fixed effects for a model including climate covariates (max temperature of the hottest month and the total precipitation of the wettest month, both over the 12 months previous to the end data of each sample), predicting either species richness (left panel) or abundance (right panel), plotted against the same fixed effects for the LUI-only model fitted in the main text. Crosses for each point represent the standard error for both the LUI + climate and LUI-only models. Here the diagonal dotted red line represents a line gradient of 1 (i.e. y=x), showing that the inclusion of climate covariates does not markedly change the predictions.

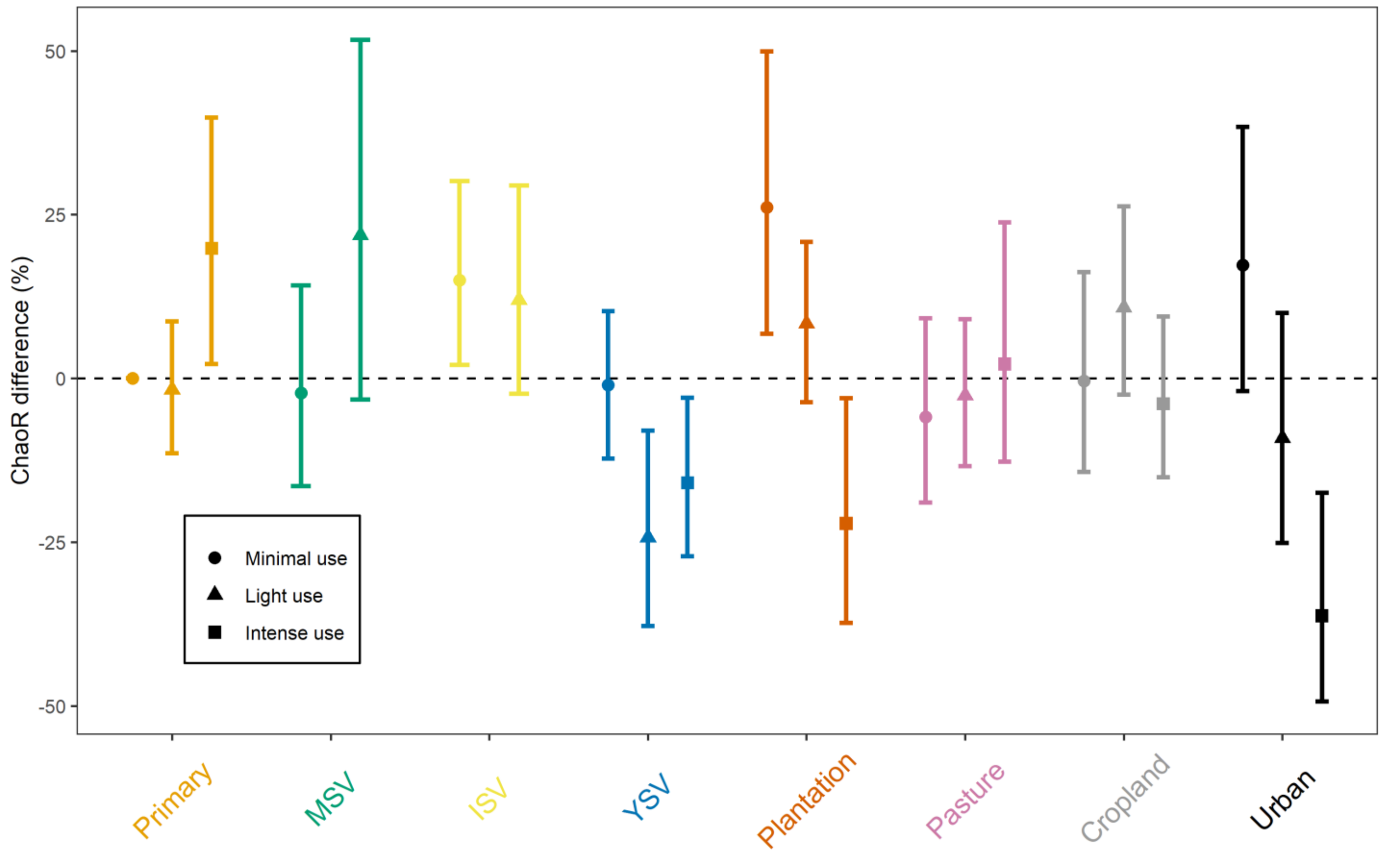

Supplementary Figure 4. Response of pollinator Chao's species richness to land-use type and land-use intensity. Colours represent land-use type: orange (primary vegetation, Primary), green (mature secondary vegetation, MSV), yellow (intermediate secondary vegetation, ISV), blue (young secondary vegetation, YSV), dark orange (plantation forest, Plantation), pink (Pasture), grey (Cropland), and black (Urban), and point shape represents land-use intensity: circle (minimal uses), triangles (light use), and squares (intense use). Effect sizes were adjusted to a percentage by drawing fixed effects 1,000 times based on the variance-covariance matrix, expressing each fixed effect as a percentage of the baseline (primary vegetation minimal use), and then calculating the median value (shown as points), and 2.5th and 97.5th percentiles (shown as error bars).

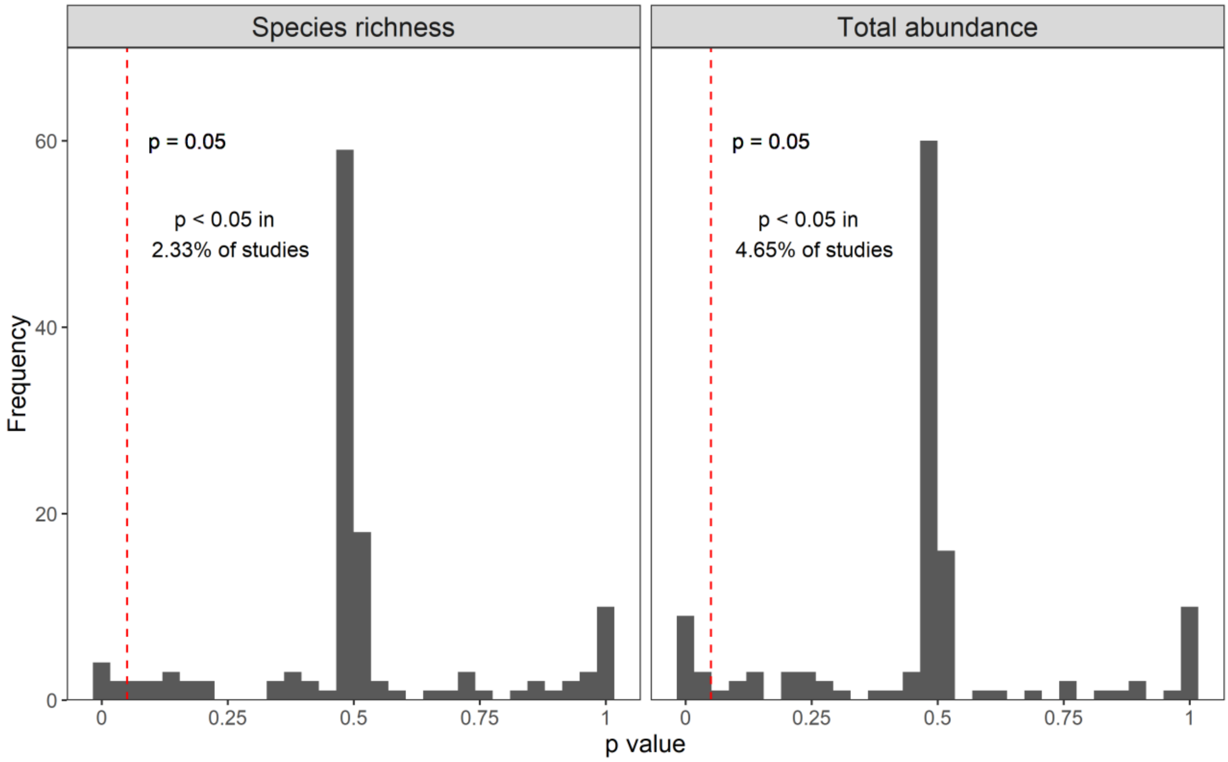

Supplementary Figure 5. The distribution of P values for a set of one-sided Moran's I tests for spatial autocorrelation, calculated for each study for species richness (left panel) and total abundance (right panel). The red dotted line in both panels represents a P value of 0.05. For species richness $p < 0.05$ in 2.33% of studies, and for total abundance $p < 0.05$ in 4.65% of studies. By chance, in the absence of spatial autocorrelation, we would expect 5% of studies to return a P value < 0.05.

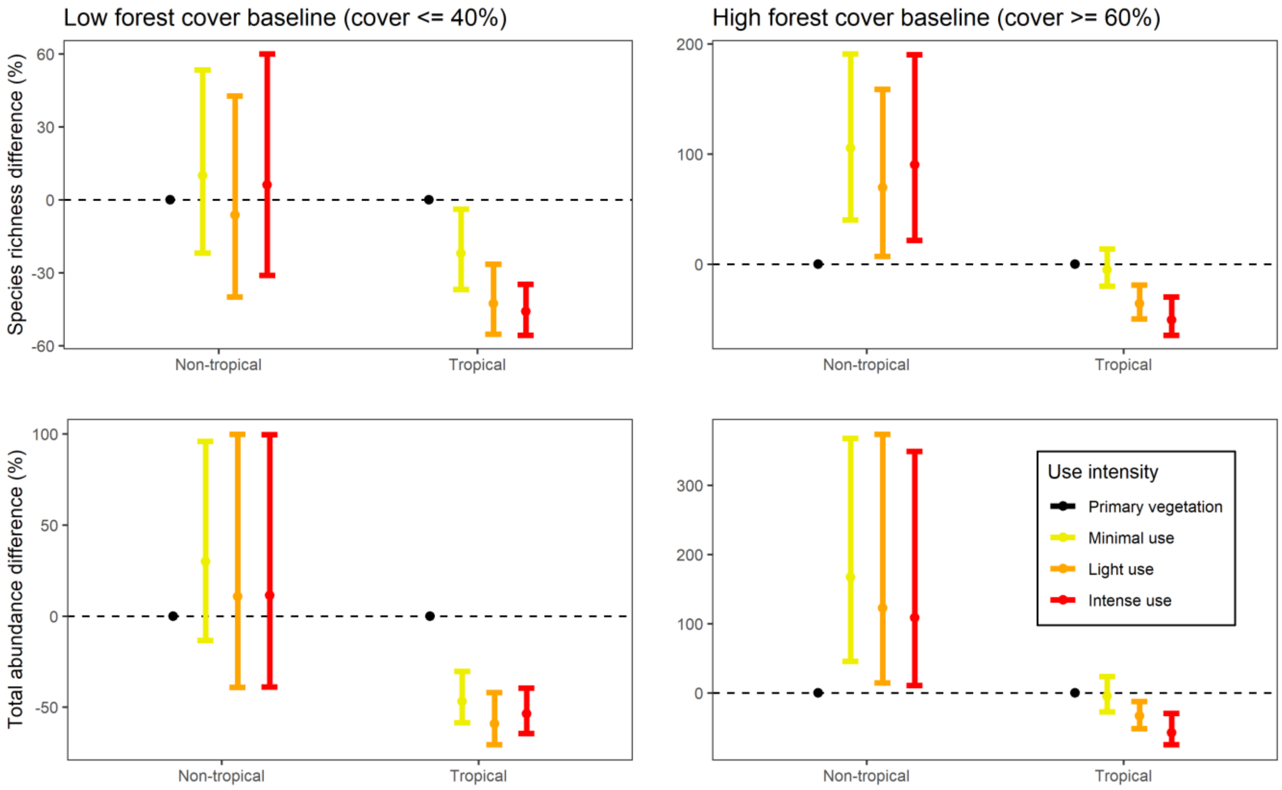

Supplementary Figure 6. Response of pollinators to land-use intensity on cropland, for non-tropical and tropical sites, when the primary vegetation minimal use baseline is shifted between high and low forest cover. Each panel represents a linear or generalised linear mixed-effects model for a given biodiversity metric. Left-hand panels (low forest cover baseline) represent species richness and total abundance predictions for cropland between tropical and non-tropical regions when the baseline is subset for only low forest cover sites (<=40% forest cover). Right-hand panels (high forest cover baseline) represent species richness and total abundance predictions for cropland when the baseline is subset for only high forest cover sites (>=60% forest cover). Effect sizes were adjusted to a percentage by sampling fixed effects 1,000 times based on the variance-covariance matrix, expressing each fixed effect as a percentage of the value in primary vegetation for that geographical zone, and then calculating the median value (shown as points), and 2.5th and 97.5th percentiles (shown as error bars).

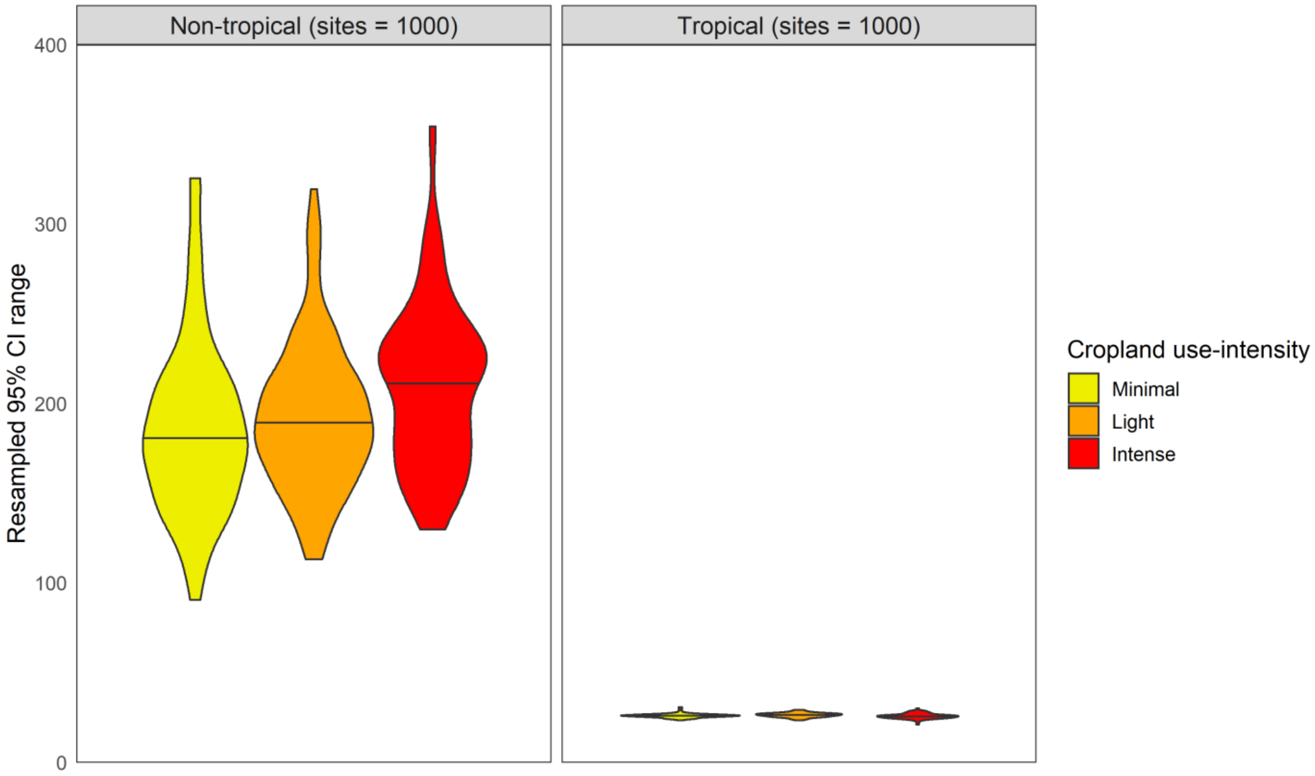

Supplementary Figure 7. Resampled 95% confidence interval ranges for total abundance for tropical and non-tropical sites. 1000 sites were resampled from each of the tropical and non-tropical sites a total of 100 times, and then for each group of 2000 (tropical and non-tropical), total abundance was fitted as a function of land-use intensity, geographical zone, and their interaction. Each violin represents the distribution of the 95% confidence interval size for all samples in each land-use intensity—geographical zone combination (the black line represents the median for all samples), indicating that there is greater variation in non-tropical responses even when sample size is controlled. For tropical sites, the distribution of the violin is close to the median since total site number (from which the sample is taken) is 1052, meaning each re-sample for the tropics is effectively all sites.

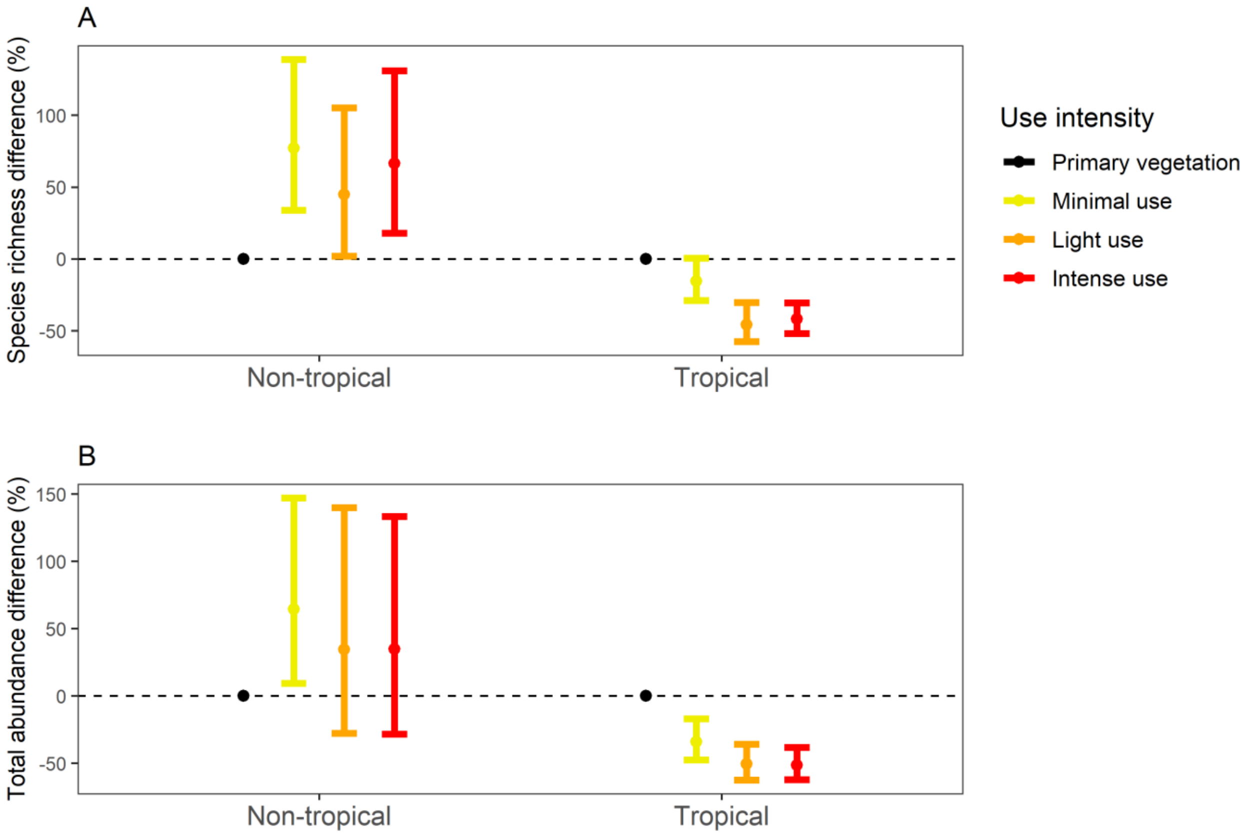

Supplementary Figure 8. Response of the main crop pollinators (bees, wasps, beetles, thrips, flies, birds, and bats) to land-use intensity on cropland, for non-tropical and tropical sites. Each panel represents a linear or generalised linear mixed-effects model for a given biodiversity metric: A, species richness; and B, total abundance. Colours represent the land-use intensity level, with primary vegetation (minimal use) as the reference factor: black (primary vegetation, minimal use); yellow (cropland, minimal use), orange (cropland, light use), and red (cropland, intense use). Effect sizes were adjusted to a percentage by sampling fixed effects 1,000 times based on the variance-covariance matrix, expressing each fixed effect as a percentage of the value in primary vegetation for that geographical zone, and then calculating the median value (shown as points), and 2.5th and 97.5th percentiles (shown as error bars).

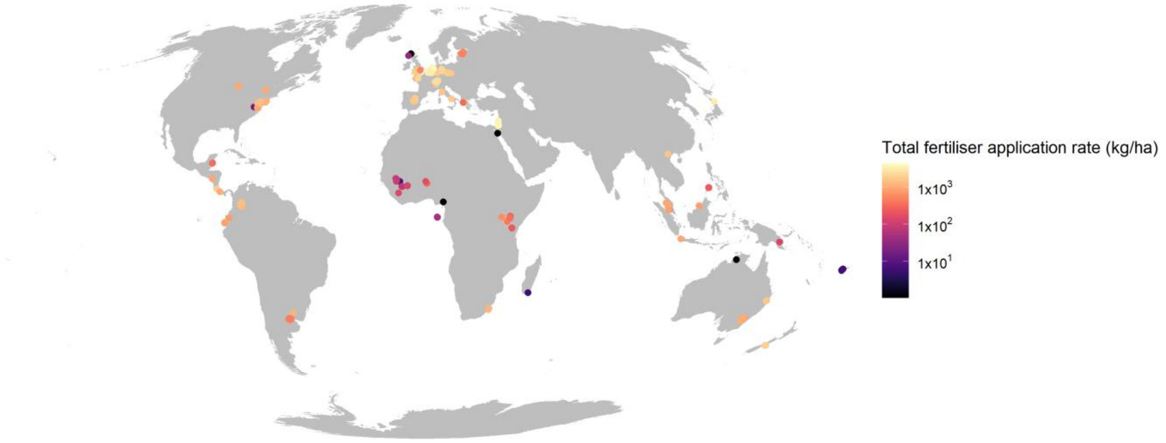

Supplementary Figure 9. Site level total fertiliser application rate (+1) for cropland sites in PREDICTS that contain pollinating species, a pair of geographical coordinates, and a fertiliser application rate for that coordinate (n=1560).

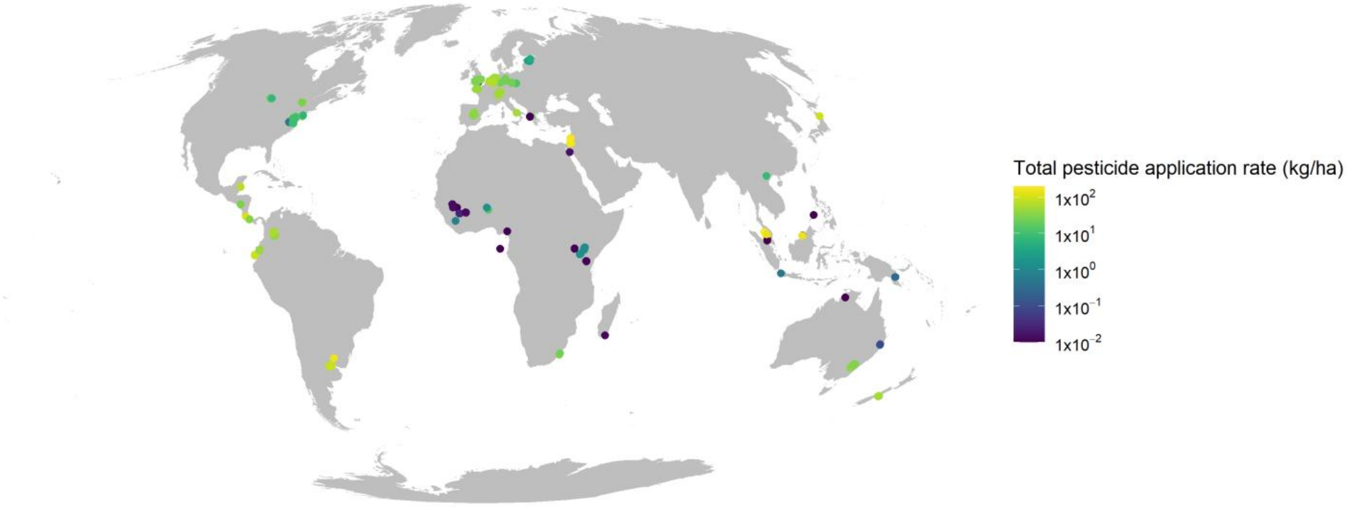

Supplementary Figure 10. Site level total pesticide application rate (+0.01) for cropland sites in PREDICTS that contain pollinating species, a pair of geographical coordinates, and a pesticide application rate for that coordinate (n=1560).

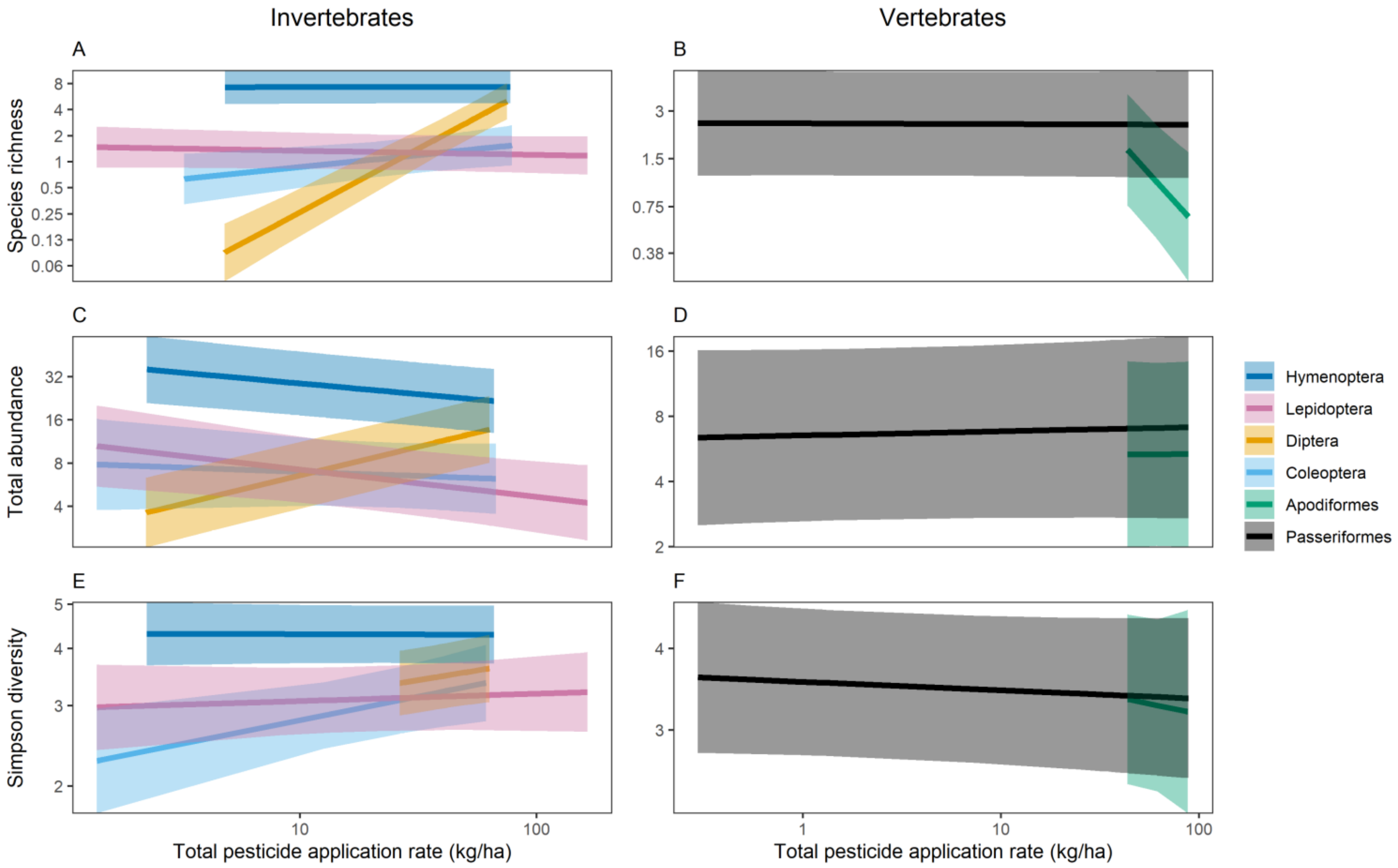

Supplementary Figure 11. Response of pollinator biodiversity in cropland sites to total pesticide application rate in the landscape (kg/ha) (note that each metric is plotted on an absolute value log scale), predicted across 95% of the range of pesticide values for each taxonomic order. Each panel represents a linear or generalised linear mixed-effects model for a given biodiversity metric for four invertebrate orders: A, C, and E, invertebrate species richness, total abundance, and Simpson diversity respectively; and B, D, and F vertebrate species richness, total abundance, and Simpson diversity respectively. Coloured lines represent median fitted estimates for each taxonomic order, with shading representing 95% confidence intervals: light blue (Coleoptera), light orange (Diptera), dark blue (Hymenoptera), pink (Lepidoptera), green (Apodiformes), and black (Passeriformes). Each biodiversity metric is predicted across 95% of the range of its values, meaning each response is not estimated across the same range of total pesticide application rate.

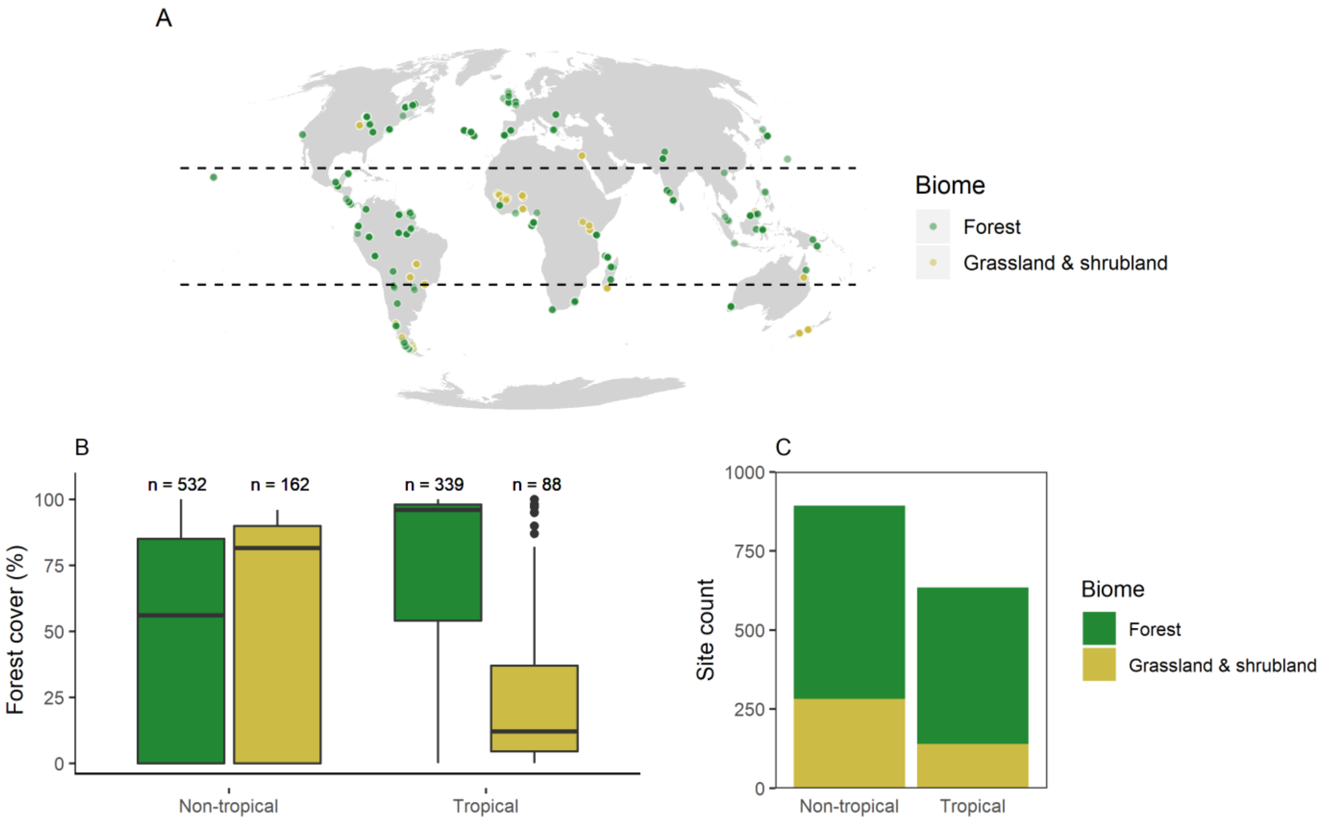

Supplementary Figure 12. The structure of primary vegetation minimal use intensity sites in PREDICTS that contain pollinating species (i.e. the baseline for Figures 2, 3, and 4). A) The global geographic distribution of' 'forest' (green) and 'grassland & shrubland' (yellow) sites according to the terrestrial ecoregions of the world (Olson et al 2001). The dashed lines represent latitudes of 23.5 and -23.5, which divide tropical from non-tropical regions. B) Boxplots for the distribution of forest cover (Hansen et al 2013) between 'grassland' and 'forest & shrubland' sites in the tropical and non-tropical zones. Here the box extends from the 25th to the 75th percentiles, the dark black inner line corresponds to the median, the upper and lower whiskers to 1.5 x IQR, and black dots to any sites beyond 1.5 x IQR. Sample size number for each grouping is represented above each boxplot. C) The frequency of 'forest' and 'grassland & shrubland' sites between the tropical and non-tropical zones.

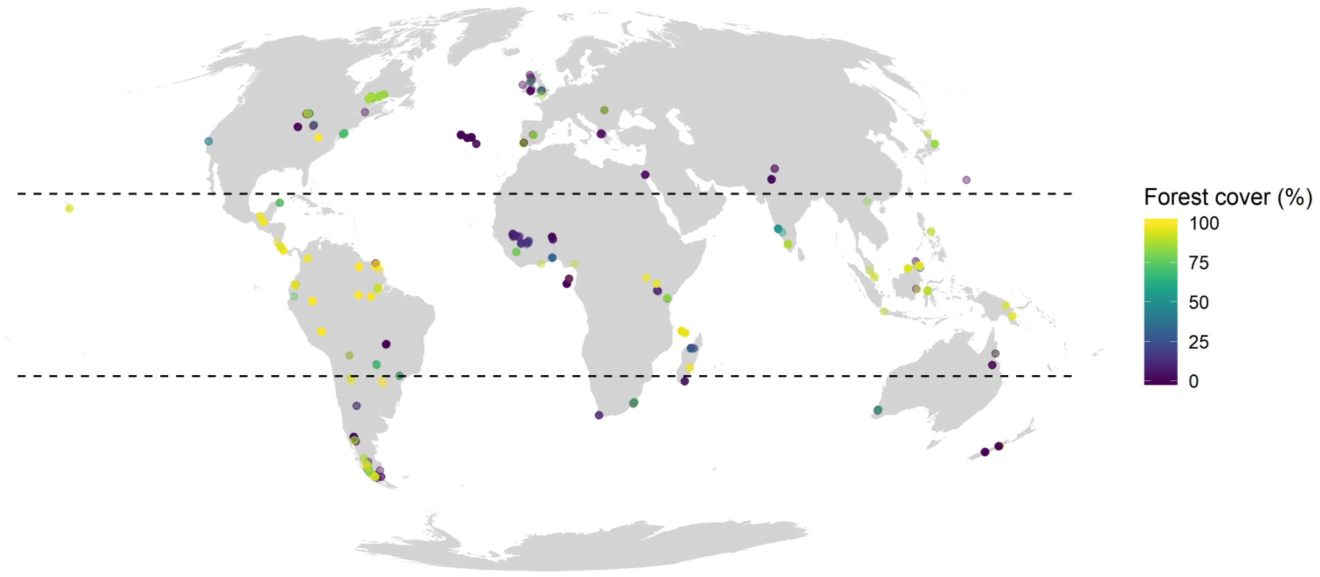

Supplementary Figure 13. The global geographic distribution of forest cover (Hansen et al 2013) for primary vegetation minimal use intensity sites in PREDICTS that contain pollinating species. Here the dashed lines represent latitudes of 23.5 and -23.5, which divide tropical from non-tropical regions.

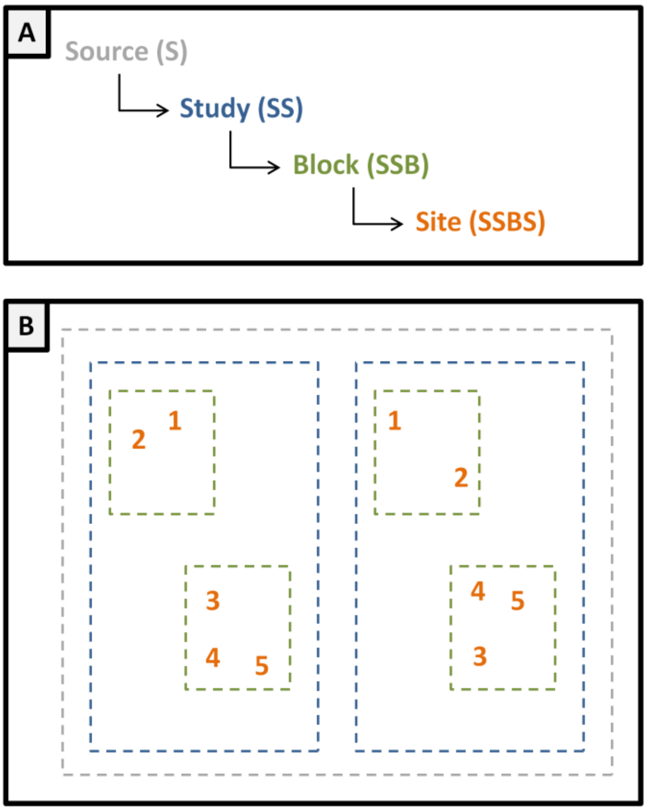

Supplementary Figure 14. Schematic of the nested structure of the PREDICTS database. A) The PREDICTS database is nested at four levels: 'source', 'study', 'block', and 'site', represented in the database as the columns S, SS, SSB, and SSBS. A source represents a unique paper, a study an experiment within a source that has a consistent sampling methodology, a block a collection of sites in a distinct spatial cluster, and site a geographic location at which biodiversity was sampled. B) The structure of the PREDICTS database is such that there can be multiple studies, blocks, and sites nested within each other.

Supplementary Tables

Supplementary Table 1. List of the 7 pollination ecologists consulted as a check-and-balance on our approach for identifying pollinating species in the PREDICTS database.

Pollination ecology expert
Opeyemi Adedoja
Sabrina Gavini
Esther Kioko
Michael Kuhlmann
Jeff Ollerton
Zong-Xin Ren
Manu Saunders

Supplementary Table 2. The total number of animal pollinating species in the PREDICTS database, aggregated by taxonomic order and class (see Figure 1).

Order	Class	N (species)
Lepidoptera	Insecta	2398
Hymenoptera	Insecta	988
Passeriformes	Aves	412
Coleoptera	Insecta	247
Diptera	Insecta	228
Apodiformes	Aves	99
Chiroptera	Mammalia	51
Psittaciformes	Aves	23
Soricomorpha	Mammalia	13
Primates	Mammalia	11
Dasyuromorphia	Mammalia	7
Rodentia	Mammalia	6
Squamata	Reptilia	5
Columbiformes	Aves	4
Didelphimorphia	Mammalia	3
Diprotodontia	Mammalia	3
Macroscelidae	Mammalia	2
Scandentia	Mammalia	1
Thysanoptera	Insecta	1

Supplementary Table 3. ANOVA tables for species richness, log(total abundance + 1), and log(Simpson diversity + 1) predicted as a function of land use-type, land-use intensity, and their interaction, and the random intercepts study (SS), block (SSB), and site (SSBS). Statistics are for either a two-sided mixed-effects generalised linear model (species richness) or a two-sided mixed-effects linear model (total abundance and species richness). *P* values are not included here for species richness since deriving these for mixed effects generalised linear models is problematic.

Response variable	Explanatory variable	df	Sum Sq	Mean Sq	F value	P value
Species richness	Land-use type	7	62.608	8.9440	8.9440	-
	Land-use intensity	2	18.877	9.4384	9.4384	-
	Type * Intensity	12	99.648	8.3040	8.3040	-
Total abundance	Land-use type	7	32.173	4.5962	8.0346	9.196e-10
	Land-use intensity	2	5.500	2.7502	4.8075	0.008192
	Type * Intensity	12	80.646	6.7205	11.7481	< 2.2e-16
Simpson diversity	Land-use type	7	2.6035	0.37193	4.4150	6.648e-05
	Land-use intensity	2	1.9661	0.98303	11.6691	8.749e-06
	Type * Intensity	12	4.6201	0.38501	4.5703	2.122e-07

Supplementary Table 4. AIC values for all the models presented in figures 2, 3, 4, and 5, as well as the insignificant model fitting fertiliser and geographical zone as an interaction. Models in which AIC is lowest are indicated by †. Significant full models are highlighted in bold.

Figure	Fixed effect structure	AIC
**LUI only models (Figure 2)**	**Richness ~ LUI**	33734.62 †
	Richness ~ 1	33871.57
	**Abundance ~ LUI**	20110.66 †
	Abundance ~ 1	20237.72
	Diversity ~ LUI	3698.13
	Diversity ~ 1	3641.20 †
**LUI * zone models (Figure 3)**	**Richness ~ LUI * zone**	13318.63 †
	Richness ~ LUI	13333.56
	Richness ~ 1	13375.75
	**Abundance ~ LUI * zone**	8093.51 †
	Abundance ~ LUI	8101.58
	Abundance ~ 1	8126.23
	Diversity ~ LUI * zone	1841.00
	Diversity ~ LUI	1830.72 †
	Diversity ~ 1	1843.73
**Fertiliser * zone models (no main text figure)**	Richness ~ fertiliser * zone	12757.00
	Richness ~ fertiliser	12756.18 †
	Richness ~ 1	12833.61
	Abundance ~ fertiliser * zone	9903.95
	Abundance ~ fertiliser	9902.60 †
	Abundance ~ 1	9967.06
	Diversity ~ fertiliser * zone	1293.94
	Diversity ~ fertiliser	1285.62 †
	Diversity ~ 1	1289.99

**LUI * order models (Figure 4)**	**Richness ~ LUI * order**	14838.27 †
	Richness ~ LUI	15957.05
	Richness ~ 1	16002.50
	**Abundance ~ LUI * order**	10604.53 †
	Abundance ~ LUI	11132.19
	Abundance ~ 1	11156.47
	**Diversity ~ LUI * order**	1808.68 †
	Diversity ~ LUI	1817.79
	Diversity ~ 1	1829.32
**Fertiliser * order models (Figure 5)**	**Richness ~ fertiliser * order**	10520.86 †
	Richness ~ fertiliser	11599.57
	Richness ~ 1	11598.16
	**Abundance ~ fertiliser * order**	8034.78 †
	Abundance ~ fertiliser	8508.81
	Abundance ~ 1	8505.94
	**Diversity ~ fertiliser * order**	1325.33 †
	Diversity ~ fertiliser	1345.22
	Diversity ~ 1	1339.64

Supplementary Table 5. Overall site representation for Figure 2, the overall response of pollinator biodiversity to LUI (land-use intensity and land-use type combined). The land-use types categories are as follows: PV (Primary vegetation), MSV (mature secondary vegetation), ISV (intermediate secondary vegetation), YSV (young secondary vegetation), PF (plantation forest) P (pasture), C (cropland), and U (urban). The intensity categories are as follows: MU (minimal use), LU (light use), and IU (intense use).

LUI	N (sites)
PVMU	1560
PVLU	996
PVIU	381
MSVMU	212
MSVLU	179
ISVMU	276
ISVLU	155
YSVMU	312
YSVLU	97
YSVIU	260
PFMU	214
PFLU	674
PFIU	110
PMU	446
PLU	532
PIU	122
CMU	426
CLU	649
CIU	658
UMU	173
ULU	136
UIU	43

Supplementary Table 6. Model summary for species richness predicted as a function of a combined factor for land-use intensity and type (LUI) and the random intercepts study (SS), block (SSB), and site (SSBS) with a two-sided mixed-effects generalised linear model (see Figure 2 for predicted values).

term	estimate	std.error	statistic	p.value
(Intercept)	1.32896397	0.09640307	13.7854939	3.116264e-43
LUI-PVLU	0.16226839	0.03122186	5.1972684	2.022382e-07
LUI-PVIU	0.14887082	0.05095798	2.9214428	3.484143e-03
LUI-MSVMU	0.06379620	0.05242243	1.2169638	2.236180e-01
LUI-MSVLU	0.21482279	0.06615476	3.2472766	1.165151e-03
LUI-ISVMU	0.15557023	0.04056605	3.8349856	1.255717e-04
LUI-ISVLU	0.20433055	0.05091357	4.0132829	5.988009e-05
LUI-YSVMU	0.02190840	0.03809628	0.5750797	5.652374e-01
LUI-YSVLU	-0.11002557	0.08427108	-1.3056149	1.916835e-01
LUI-YSVIU	-0.17073604	0.05050497	-3.3805792	7.233322e-04
LUI-PFMU	0.24269714	0.05451398	4.4520166	8.506757e-06
LUI-PFLU	0.05463885	0.03714311	1.4710359	1.412814e-01
LUI-PFIU	-0.11853355	0.06879615	-1.7229678	8.489436e-02
LUI-PMU	0.15678740	0.04408967	3.5561029	3.763967e-04
LUI-PLU	-0.03122227	0.04119039	-0.7579990	4.484516e-01
LUI-PIU	0.01590149	0.06782871	0.2344359	8.146466e-01
LUI-CMU	-0.01540370	0.04786049	-0.3218458	7.475695e-01
LUI-CLU	-0.11717122	0.05037602	-2.3259323	2.002216e-02
LUI-CIU	-0.13700839	0.04998200	-2.7411547	6.122367e-03
LUI-UMU	0.23228075	0.06259899	3.7106150	2.067564e-04
LUI-ULU	0.03215269	0.06336714	0.5074032	6.118719e-01
LUI-UIU	-0.22080354	0.08463465	-2.6089024	9.083313e-03

Supplementary Table 7. Model summary for log(total abundance + 1) predicted as a function of a combined factor for land-use intensity and type (LUI) and the random intercepts study (SS) and block (SSB) with a two-sided mixed-effects linear model (see Figure 2 for predicted values).

term	estimate	std.error	statistic	p.value
(Intercept)	3.066663564	0.13047422	23.50398104	1.441767e-66
LUI-PVLU	0.328309492	0.04936891	6.65012621	3.125781e-11
LUI-PVIU	0.371272193	0.07205361	5.15272138	2.631861e-07
LUI-MSVMU	0.026126195	0.08501411	0.30731599	7.586111e-01
LUI-MSVLU	0.361740682	0.09563360	3.78256885	1.563882e-04
LUI-ISVMU	0.217422866	0.07366012	2.95170376	3.169719e-03
LUI-ISVLU	0.310991569	0.07822650	3.97552697	7.089066e-05
LUI-YSVMU	0.149413495	0.07011363	2.13101940	3.311893e-02
LUI-YSVLU	-0.008061529	0.13255730	-0.06081543	9.515078e-01
LUI-YSVIU	0.110668437	0.08303010	1.33287130	1.826132e-01
LUI-PFMU	0.362561497	0.08599708	4.21597432	2.516897e-05
LUI-PFLU	0.063777193	0.06055574	1.05319819	2.922826e-01
LUI-PFIU	0.189445958	0.12859996	1.47314167	1.407558e-01
LUI-PMU	0.584565062	0.05851292	9.99035868	2.329859e-23
LUI-PLU	0.254135112	0.05423478	4.68583282	2.837077e-06
LUI-PIU	0.041868650	0.10917168	0.38351202	7.013506e-01
LUI-CMU	0.006691476	0.06373908	0.10498232	9.163925e-01
LUI-CLU	-0.066291107	0.07378357	-0.89845354	3.689713e-01
LUI-CIU	-0.217808927	0.07491060	-2.90758476	3.653874e-03
LUI-UMU	0.424397518	0.09029529	4.70010712	2.644965e-06
LUI-ULU	0.408046211	0.12605275	3.23710689	1.212531e-03
LUI-UIU	-0.096260227	0.16519365	-0.58271142	5.601051e-01

Supplementary Table 8. Cropland site representation for Figure 3, the overall response of pollinator biodiversity to land-use intensity between the non-tropical and tropical zones

Geographical zone	LUI	N (sites)
Non-tropical	Primary vegetation	893
Non-tropical	Minimal use cropland	245
Non-tropical	Light use cropland	492
Non-tropical	Intense use cropland	578
Tropical	Primary vegetation	634
Tropical	Minimal use cropland	181
Tropical	Light use cropland	157
Tropical	Intense use cropland	80

Supplementary Table 9. Model output for species richness predicted as a function of a combined factor for land-use intensity and type (LUI), geographical zone (tropics/non-tropics), and the random intercepts study (SS), block (SSB), and site (SSBS) with a two-sided mixed-effects generalised linear model (see Figure 3 for predicted values).

term	estimate	std.error	statistic	p.value
(Intercept)	1.2092244	0.1822545	6.634814	3.249122e-11
LUI-Minimal use	0.3735829	0.1303490	2.866020	4.156686e-03
LUI-Light use	0.1965426	0.1779420	1.104532	2.693624e-01
LUI-Intense use	0.3271289	0.1769412	1.848800	6.448668e-02
zone-Tropics	0.2388790	0.2471688	0.966461	3.338136e-01
LUI-Minimal use:zone-Tropics	-0.5307787	0.1477803	-3.591674	3.285612e-04
LUI-Light use:zone-Tropics	-0.6940822	0.2054538	-3.378289	7.293833e-04
LUI-Intense use:zone-Tropics	-0.9160952	0.1980057	-4.626611	3.716984e-06

Supplementary Table 10. Model output for log(total abundance + 1) predicted as a function of a combined factor for land-use intensity and type (LUI), geographical zone (tropics/non-tropics), and the random intercepts study (SS) and block (SSB) with a two-sided mixed-effects linear model (see Figure 3 for predicted values).

term	estimate	std.error	statistic	p.value
(Intercept)	2.7469928	0.2460986	11.1621626	8.118052e-23
LUI-Minimal use	0.4692762	0.1790959	2.6202502	8.831766e-03
LUI-Light use	0.3010843	0.2895618	1.0397928	2.990043e-01
LUI-Intense use	0.2816584	0.2848026	0.9889603	3.232719e-01
zone-Tropics	0.5182296	0.3380493	1.5330002	1.270054e-01
LUI-Minimal use:zone-Tropics	-0.8576550	0.2028786	-4.2274302	2.434102e-05
LUI-Light use:zone-Tropics	-0.9745301	0.3161887	-3.0821156	2.146717e-03
LUI-Intense use:zone-Tropics	-0.9607852	0.3106910	-3.0924140	2.083223e-03

Supplementary Table 11. Cropland site representation for Figure 4, the response of 6 taxonomic orders (Hymenoptera, Diptera, Lepidoptera, Coleoptera, Passeriformes, and Apodiformes) to land-use intensity on cropland. Note that the total number of sites for each order-intensity group will not sum to the total of 3006, since some sites are represented across multiple groups.

Taxonomic order	LUI	N (sites)
Hymenoptera	Primary vegetation	186
Hymenoptera	Minimal use cropland	180
Hymenoptera	Light use cropland	564
Hymenoptera	Intense use cropland	578
Diptera	Primary vegetation	25
Diptera	Minimal use cropland	50
Diptera	Light use cropland	91
Diptera	Intense use cropland	72
Lepidoptera	Primary vegetation	317
Lepidoptera	Minimal use cropland	76
Lepidoptera	Light use cropland	112
Lepidoptera	Intense use cropland	44
Coleoptera	Primary vegetation	342
Coleoptera	Minimal use cropland	90
Coleoptera	Light use cropland	112
Coleoptera	Intense use cropland	44
Passeriformes	Primary vegetation	637
Passeriformes	Minimal use cropland	120
Passeriformes	Light use cropland	53
Passeriformes	Intense use cropland	61
Apodiformes	Primary vegetation	108
Apodiformes	Minimal use cropland	33
Apodiformes	Light use cropland	20

Supplementary Table 12. Model output for species richness predicted as a function of taxonomic order and a combined factor for land-use intensity and type (LUI), and the random intercepts study (SS), block (SSB), and site (SSBS) with a two-sided mixed-effects generalised linear model (see Figure 4 for predicted values).

term	estimate	std.error	statistic	p.value
(Intercept)	2.20840153	0.1991447	11.0894288	1.411863e-28
Order-Diptera	-0.83341036	0.1530930	-5.4438158	5.215114e-08
Order-Lepidoptera	-1.13968603	0.1506288	-7.5661918	3.843246e-14
Order-Coleoptera	-1.55508820	0.1592342	-9.7660458	1.574787e-22
Order-Passeriformes	-1.14836510	0.2985974	-3.8458648	1.201280e-04
Order-Apodiformes	-0.93317182	0.3072722	-3.0369547	2.389814e-03
LUI-Minimal use	0.37282903	0.2126349	1.7533761	7.953747e-02
LUI-Light use	-0.17614884	0.1930156	-0.9126147	3.614452e-01
LUI-Intense use	-0.04344631	0.1967355	-0.2208362	8.252200e-01
Order-Diptera:LUI-Minimal use	-3.18780581	0.4359546	-7.3122431	2.627192e-13
Order-Lepidoptera:LUI-Minimal use	-0.69525855	0.2226143	-3.1231526	1.789249e-03
Order-Coleoptera:LUI-Minimal use	-0.59864325	0.2378589	-2.5168001	1.184260e-02
Order-Passeriformes:LUI-Minimal use	-0.15672200	0.2441114	-0.6420102	5.208665e-01
Order-Apodiformes:LUI-Minimal use	-0.54035561	0.2681862	-2.0148525	4.392011e-02
Order-Diptera:LUI-Light use	-0.18953673	0.1934202	-0.9799221	3.271246e-01
Order-Lepidoptera:LUI-Light use	-0.54235377	0.1986485	-2.7302187	6.329232e-03
Order-Coleoptera:LUI-Light use	-0.48525618	0.2173994	-2.2320957	2.560864e-02
Order-Passeriformes:LUI-Light use	0.07107473	0.2844426	0.2498737	8.026850e-01
Order-Apodiformes:LUI-Light use	-0.77090778	0.3367013	-2.2895892	2.204514e-02
Order-Diptera:LUI-Intense use	-0.91597731	0.1926870	-4.7537046	1.997228e-06
Order-Lepidoptera:LUI-Intense use	-0.91324565	0.2345930	-3.8928932	9.905577e-05
Order-Coleoptera:LUI-Intense use	-0.27001790	0.2304695	-1.1715992	2.413580e-01
Order-Passeriformes:LUI-Intense use	-0.40540007	0.2270651	-1.7853916	7.419781e-02

Supplementary Table 13. Model output for log(total abundance + 1) predicted as a function of taxonomic order and a combined factor for land-use intensity and type (LUI), and the random intercepts study (SS) and block (SSB) with a two-sided mixed-effects linear model (see Figure 4 for predicted values).

term	estimate	std.error	statistic	p.value
(Intercept)	4.73922131	0.2829571	16.7489059	6.296397e-47
Order-Diptera	-1.44912912	0.2550315	-5.6821574	1.438105e-08
Order-Lepidoptera	-1.73354917	0.2270221	-7.6360361	2.999194e-14
Order-Coleoptera	-1.80793239	0.2292210	-7.8872900	4.291811e-15
Order-Passeriformes	-2.30268897	0.4118455	-5.5911479	7.372749e-08
Order-Apodiformes	-2.11948413	0.4227288	-5.0138155	1.094958e-06
LUI-Minimal use	-1.10153763	0.2873542	-3.8333794	1.291541e-04
LUI-Light use	-1.69955568	0.2656710	-6.3972204	1.875913e-10
LUI-Intense use	-1.40296862	0.2701440	-5.1934097	2.223549e-07
Order-Diptera:LUI-Minimal use	-0.99823141	0.3047054	-3.2760539	1.062431e-03
Order-Lepidoptera:LUI-Minimal use	0.12833239	0.2894333	0.4433920	6.575106e-01
Order-Coleoptera:LUI-Minimal use	0.43580445	0.2891418	1.5072346	1.318455e-01
Order-Passeriformes:LUI-Minimal use	1.32596000	0.3318642	3.9954900	6.598938e-05
Order-Apodiformes:LUI-Minimal use	0.92948978	0.3435239	2.7057499	6.848714e-03
Order-Diptera:LUI-Light use	1.03211340	0.2820608	3.6591877	2.566325e-04
Order-Lepidoptera:LUI-Light use	0.39551699	0.2543970	1.5547232	1.201104e-01
Order-Coleoptera:LUI-Light use	0.44574567	0.2562287	1.7396399	8.201790e-02
Order-Passeriformes:LUI-Light use	1.69308440	0.3458009	4.8961252	1.025282e-06
Order-Apodiformes:LUI-Light use	0.98890956	0.3481864	2.8401732	4.536227e-03
Order-Diptera:LUI-Intense use	-0.18244690	0.2881395	-0.6331894	5.266497e-01
Order-Lepidoptera:LUI-Intense use	-0.16831841	0.2791143	-0.6030447	5.465186e-01
Order-Coleoptera:LUI-Intense use	0.05306171	0.2808995	0.1888993	8.501829e-01
Order-Passeriformes:LUI-Intense use	1.05068214	0.3225579	3.2573444	1.136272e-03

Supplementary Table 14. Model output for log(Simpson diversity + 1) predicted as a function of taxonomic order and a combined factor for land-use intensity and type (LUI), and the random intercepts study (SS) and block (SSB) with a two-sided mixed-effects linear model (see Figure 4 for predicted values).

term	estimate	std.error	statistic	p.value
(Intercept)	1.58383270	0.09627049	16.4519030	2.162291e-45
Order-Diptera	-0.06544099	0.09414709	-0.6950930	4.870580e-01
Order-Lepidoptera	-0.16830797	0.08304914	-2.0266070	4.282549e-02
Order-Coleoptera	-0.27585315	0.08486779	-3.2503870	1.170032e-03
Order-Passeriformes	-0.34473774	0.13620897	-2.5309473	1.217500e-02
Order-Apodiformes	-0.25772117	0.14095948	-1.8283351	6.885026e-02
LUI-Minimal use	0.12659585	0.11562792	1.0948553	2.738108e-01
LUI-Light use	-0.08470655	0.10185990	-0.8315986	4.057828e-01
LUI-Intense use	-0.03002987	0.10306170	-0.2913776	7.708067e-01
Order-Diptera:LUI-Minimal use	-0.50068423	0.16753455	-2.9885431	2.828102e-03
Order-Lepidoptera:LUI-Minimal use	-0.09384230	0.13338019	-0.7035700	4.817930e-01
Order-Coleoptera:LUI-Minimal use	-0.20716970	0.12646894	-1.6381074	1.015956e-01
Order-Passeriformes:LUI-Minimal use	-0.02003844	0.12992647	-0.1542290	8.774498e-01
Order-Apodiformes:LUI-Minimal use	-0.10811138	0.13493663	-0.8012011	4.231267e-01
Order-Diptera:LUI-Light use	-0.15765579	0.10769809	-1.4638680	1.433469e-01
Order-Lepidoptera:LUI-Light use	-0.10832984	0.10223874	-1.0595772	2.894413e-01
Order-Coleoptera:LUI-Light use	-0.10885346	0.10298404	-1.0569935	2.906226e-01
Order-Passeriformes:LUI-Light use	0.05550724	0.13072949	0.4245962	6.711757e-01
Order-Apodiformes:LUI-Light use	-0.13518246	0.14040177	-0.9628259	3.357357e-01
Order-Diptera:LUI-Intense use	-0.11398265	0.11574556	-0.9847691	3.248249e-01
Order-Lepidoptera:LUI-Intense use	-0.26533172	0.11391675	-2.3291720	1.992766e-02
Order-Coleoptera:LUI-Intense use	0.06450120	0.11161580	0.5778859	5.633927e-01
Order-Passeriformes:LUI-Intense use	-0.26787298	0.11890101	-2.2529076	2.438168e-02

Supplementary Table 15. Cropland site representation for Figure 5, the response of 6 taxonomic orders (Hymenoptera, Diptera, Lepidoptera, Coleoptera, Passeriformes, and Apodiformes) to total fertiliser application rate (kg/ha) on cropland. Note that the total number of sites for each order-intensity group will not sum to the total of 2190, since some sites are represented across multiple groups.

**Taxonomic order**	**N (sites)**
Hymenoptera	1355
Passeriformes	683
Lepidoptera	258
Coleoptera	246
Apodiformes	244
Diptera	213

Supplementary Table 16. Model output for species richness predicted as a function of log10(fertiliser application rate), taxonomic order, and the random intercepts study (SS), block (SSB), and site (SSBS) with a two-sided mixed-effects generalised linear model (see Figure 5 for predicted values.

**term**	**estimate**	**std.error**	**statistic**	**p.value**
(Intercept)	-4.952880	1.9133878	-2.588539	9.638395e-03
log10(fert)	1.763928	0.6462196	2.729610	6.340926e-03
Order-Coleoptera	4.066850	2.1788416	1.866519	6.196874e-02
Order-Diptera	-6.581990	2.2395758	-2.938945	3.293316e-03
Order-Hymenoptera	7.782467	1.9674357	3.955640	7.633008e-05
Order-Lepidoptera	6.816010	2.1208452	3.213818	1.309829e-03
Order-Passeriformes	5.717434	1.7523151	3.262789	1.103217e-03
log10(fert):Order-Coleoptera	-1.397608	0.7389379	-1.891374	5.857445e-02
log10(fert):Order-Diptera	2.184480	0.7458250	2.928945	3.401149e-03
log10(fert):Order-Hymenoptera	-2.044201	0.6595253	-3.099504	1.938449e-03
log10(fert):Order-Lepidoptera	-2.372238	0.7163021	-3.311784	9.270311e-04
log10(fert):Order-Passeriformes	-1.753834	0.5951118	-2.947067	3.208041e-03

Supplementary Table 17. Model output for log(total abundance + 1) predicted as a function of log10(fertiliser application rate), taxonomic order, and the random intercepts study (SS) and block (SSB) with a two-sided mixed-effects linear model (see Figure 5 for predicted values.

term	estimate	std.error	statistic	p.value
(Intercept)	1.54088771	1.6289045	0.94596564	3.443103e-01
log10(fert)	-0.05127043	0.5798805	-0.08841551	9.295537e-01
Order-Coleoptera	1.73923685	2.0015144	0.86896046	3.852573e-01
Order-Diptera	-8.43201071	1.9876442	-4.24221343	2.316702e-05
Order-Hymenoptera	4.91168470	1.8050664	2.72105487	6.581797e-03
Order-Lepidoptera	3.02481460	1.9954106	1.51588579	1.300592e-01
Order-Passeriformes	0.37425442	1.3446391	0.27833076	7.807799e-01
log10(fert):Order-Coleoptera	-0.41448339	0.6991881	-0.59280667	5.534798e-01
log10(fert):Order-Diptera	3.07974048	0.6879237	4.47686346	7.887758e-06
log10(fert):Order-Hymenoptera	-0.99775501	0.6265387	-1.59248750	1.114068e-01
log10(fert):Order-Lepidoptera	-0.88228527	0.6962327	-1.26722749	2.053937e-01
log10(fert):Order-Passeriformes	-0.03415814	0.4917438	-0.06946330	9.446260e-01

Supplementary Table 18. Model output for log(Simpson diversity + 1) predicted as a function of log10(fertiliser application rate), taxonomic order, and the random intercepts study (SS) and block (SSB) with a two-sided mixed-effects linear model (see Figure 5 for predicted values.

term	estimate	std.error	statistic	p.value
(Intercept)	0.31314652	0.9728151	0.32189726	0.7475667
log10(fert)	0.31316180	0.3390191	0.92372904	0.3557465
Order-Coleoptera	0.06297832	1.0489090	0.06004174	0.9521328
Order-Diptera	-0.75504668	1.5538186	-0.48592974	0.6270741
Order-Hymenoptera	1.39980252	1.0140713	1.38037886	0.1676404
Order-Lepidoptera	1.25212031	1.0516298	1.19064739	0.2339989
Order-Passeriformes	0.78924098	0.9157879	0.86181636	0.3889024
log10(fert):Order-Coleoptera	-0.05337955	0.3650140	-0.14623974	0.8837564
log10(fert):Order-Diptera	0.23220352	0.5204045	0.44619817	0.6555056
log10(fert):Order-Hymenoptera	-0.39618381	0.3504534	-1.13048915	0.2584168
log10(fert):Order-Lepidoptera	-0.46442622	0.3650117	-1.27235982	0.2034519
log10(fert):Order-Passeriformes	-0.26699620	0.3178015	-0.84013513	0.4009433

Supplementary Table 19. Pseudo R squared for all main models included in the main text.

	Model	Conditional pseudo R squared	Marginal pseudo R squared
**Figure 2**	Richness ~ LUI	0.706	0.004
	Abundance ~ LUI	0.876	0.009
**Figure 3**	Richness ~ LUI * zone	0.714	0.009
	Abundance ~ LUI * zone	0.887	0.010
**Figure 4**	Richness ~ LUI * order	0.753	0.149
	Abundance ~ LUI * order	0.850	0.094
	Diversity ~ LUI * order	0.828	0.055
**Figure 5**	Richness ~ fertiliser * order	0.775	0.169
	Abundance ~ fertiliser * order	0.841	0.114
	Diversity ~ fertiliser * order	0.700	0.058

Supplementary Table 20. Crops for which fertiliser application rate estimates are available in the Earthstat data, each with estimates of nitrogen, phosphorus, and potassium.

**Crop**
Barley
Cassava
Cotton
Groundnut
Maize
Millet
Oilpalm
Potato
Rapeseed
Rice
Rye
Sorghum
Soybean
Sugarbeet
Sugarcane
Sunflower
Wheat
