# Supplementary material for: Global effects of land-use intensity on local pollinator biodiversity
Source: Nat Commun. 2021 May 18;12:2902. doi: 10.1038/s41467-021-23228-3 (PMC8131357; doi:10.1038/s41467-021-23228-3)
Supplement: Supplementary file 3 — Description of Additional Supplementary Files [file 41467_2021_23228_MOESM3_ESM.docx]

**Description of Additional Supplementary Files**

File name: Supplementary Data 1

Description: Expert assessment csv for all potentially pollinating genera in the PREDICTS database. Each row represents an animal genus. The column ‘status’ indicates whether a genus was ‘kept, ‘excluded’, or ‘added’ following consultation with pollination ecology experts. ‘KEPT’ indicates any genus initially identified as a pollinator which a panel of experts confirmed, ‘EXCLUDED’ indicates any genus initially identified as a pollinator which a panel of experts suggested to remove, and ‘ADDED’ indicates any genus not initially identified as a pollinator that a panel of experts suggested should be included.

File name: Supplementary Data 2

Description: A list of references used in the initial confirmation of likely pollinating species. ‘Title’ indicates the title of each text, and ‘Author (year)’ the author and year of publication for each text.
