# Supplementary material for: Global effects of land-use intensity on local pollinator biodiversity
Source: Nat Commun. 2021 May 18;12:2902. doi: 10.1038/s41467-021-23228-3 (PMC8131357; doi:10.1038/s41467-021-23228-3)
Supplement: Supplementary file 6 — Reporting Summary [file 41467_2021_23228_MOESM6_ESM.pdf]

nature research

Corresponding author(s): Joseph Millard

Last updated by author(s): Mar 9, 2021

Reporting Summary

Nature Research wishes to improve the reproducibility of the work that we publish. This form provides structure for consistency and transparency in reporting. For further information on Nature Research policies, see our Editorial Policies and the Editorial Policy Checklist.

Statistics

For all statistical analyses, confirm that the following items are present in the figure legend, table legend, main text, or Methods section.

n/a	Confirmed	
☐	☒	The exact sample size (n) for each experimental group/condition, given as a discrete number and unit of measurement
☒	☐	A statement on whether measurements were taken from distinct samples or whether the same sample was measured repeatedly
☐	☒	The statistical test(s) used AND whether they are one- or two-sided<br>*Only common tests should be described solely by name; describe more complex techniques in the Methods section.*
☐	☒	A description of all covariates tested
☐	☒	A description of any assumptions or corrections, such as tests of normality and adjustment for multiple comparisons
☐	☒	A full description of the statistical parameters including central tendency (e.g. means) or other basic estimates (e.g. regression coefficient) AND variation (e.g. standard deviation) or associated estimates of uncertainty (e.g. confidence intervals)
☐	☒	For null hypothesis testing, the test statistic (e.g. F, t, r) with confidence intervals, effect sizes, degrees of freedom and P value noted<br>*Give P values as exact values whenever suitable.*
☒	☐	For Bayesian analysis, information on the choice of priors and Markov chain Monte Carlo settings
☐	☒	For hierarchical and complex designs, identification of the appropriate level for tests and full reporting of outcomes
☐	☒	Estimates of effect sizes (e.g. Cohen's d, Pearson's r), indicating how they were calculated

Our web collection on statistics for biologists contains articles on many of the points above.

Software and code

Policy information about availability of computer code

Data collection	All data compilation and cleaning was carried out in R v4.0.3. The R package 'taxize' v0.9.97 was used to scrape taxonomic names from pollination related abstracts. All R code for the compilation and cleaning of data is publicly available on on Zenodo via GitHub (https://doi.org/10.5281/zenodo.4593493).
Data analysis	All data analysis was carried out in R v4.0.3. The R package 'StatisticalModels' v0.1 was used to test for overdispersion in the species richness models. All R code for the analysis of data is publicly available on Zenodo via GitHub (https://doi.org/10.5281/zenodo.4593493).

For manuscripts utilizing custom algorithms or software that are central to the research but not yet described in published literature, software must be made available to editors and reviewers. We strongly encourage code deposition in a community repository (e.g. GitHub). See the Nature Research guidelines for submitting code & software for further information.

Data

Policy information about availability of data

All manuscripts must include a data availability statement. This statement should provide the following information, where applicable:

- Accession codes, unique identifiers, or web links for publicly available datasets
- A list of figures that have associated raw data
- A description of any restrictions on data availability

A number of publicly available data sets were used as part of this analysis. We used the PREDICTS database for global biodiversity records of pollinating species and site-level categorical factors of land-use type and intensity (https://doi.org/10.5519/0066354). Our subset of pollinating species in the PREDICTS database is available on FigShare (https://doi.org/10.6084/m9.figshare.12815669.v2). We used EarthStat fertiliser application rate data to calculate site-level total fertiliser application rate for the years 1999-2000 (https://doi.org/10.1038/nature11420 & https://doi.org/10.1126/science.1246067). We used WorldClim 2.1 monthly average maximum temperature and monthly total precipitation to calculate potentially confounding climatic variables (https://www.worldclim.org/data/

index.html). We used global forest cover data from Hansen et al to test the effect of low and high forest cover baselines (https://doi.org/10.1126/science.1244693). We used PEST-CHEMGRIDS to calculate site-level total pesticide application rate (https://doi.org/10.1038/s41597-019-0169-4).

Field-specific reporting

Please select the one below that is the best fit for your research. If you are not sure, read the appropriate sections before making your selection.

- [ ] Life sciences
- [ ] Behavioural & social sciences
- [x] Ecological, evolutionary & environmental sciences

For a reference copy of the document with all sections, see nature.com/documents/nr-reporting-summary-flat.pdf

Ecological, evolutionary & environmental sciences study design

All studies must disclose on these points even when the disclosure is negative.

Study description	Using mixed effects models we modeled the response of local pollinator biodiversity (total abundance, species richness, and Simpson diversity) to a categorical factor of land-use intensity and fertiliser application rate.
Research sample	We extracted records of pollinator biodiversity from the PREDICTS database. Records of pollinator abundance were identified through searching the literature and consulting experts, and then subsetting these species from PREDICTS. The PREDICTS database contains records of local species abundance for the globe, recorded in both natural and anthropogenic land-use types. PREDICTS is structured hierarchically, with each abundance measurement nested at four levels: Source, Study, Block, Site.
Sampling strategy	We used the publicly available PREDICTS database to extract abundance values for pollinating species.
Data collection	The PREDICTS database is publicly available, meaning no data collection was required for our biodiversity measurements. Fertiliser application rate data was downloaded from EARTHSTAT (http://www.earthstat.org/nutrient-application-major-crops/), and then summed for all fertiliser-crop combinations. In a set of additional analyses we also used PEST CHEMGRIDS (https://doi.org/10.1038/s41597-019-0169-4) for global estimates of pesticide application rate, WorldClim for max temperature and precipitation (https://www.worldclim.org/data/index.html), and Hansen et al for global forest cover data (https://earthenginepartners.appspot.com/science-2013-global-forest/download_v1.2.html). The fertiliser data was compiled by Charlotte Outhwaite and the forest cover data by Monica Ortiz. All other datasets were compiled by Joseph Millard.
Timing and spatial scale	Our pollinator subset of the PREDICTS database is global in spatial extent. Abundance measurements in the PREDICTS database are from 1984-2003, with 95% made since 2000. PREDICTS measurements have varying start and stop dates of data collection, recorded in the database for each measurement. EARTHSTAT fertiliser application rate data mostly represents the year 2000, going back as far as 1994 and as recent as 2001, at a spatial scale of ~10km (at the equator). WorldClim monthly climate data was extracted for the month of the end date of each PREDICTS sample and 11 months previous, at a spatial scale of ~4.5km. Hansen et al forest cover data is for the year 2000 for all vegetation taller than 5m in height, at a spatial scale of ~30m.
Data exclusions	For our initial analysis of overall response to land-use intensity we removed categories with low site representation (see Methods), since at low site number we could not make robust predictions of pollinator response to intensity. We made a decision to remove categories prior to analysis, but could only set thresholds for exclusion after observing site distribution among categories. For all subsequent analyses we focused specifically on cropland sites. We made a prior decision to focus on cropland given the importance of animal pollination to crop production. For any analysis examining differences among taxonomic groups we removed any taxonomic group with low site representation (see Methods).
Reproducibility	The analysis in this study is based on a collation of abundance measurements for pollinating species in the PREDICTS database. Given there is not another similar database for pollinators, we were not able to independently reproduce our results. However, we did undertake a series of validation analyses which indicate that the results of our study likely would be reproducible with an independent set of measurements: We controlled for the hierarchical structure of our data through mixed-effects models; we tested each model against a set of null models using AIC values; we tested for spatial autocorrelation of model residuals; we tested alternate model error distributions (e.g. negative binomial); we tested for the effects of potentially confounding climatic variables; we tested the effect of jack-knifing our models for continental regions; we checked the extent to which an abundance controlled measure of species richness differed from our raw measure of species richness; and we checked the extent to which shifting the forest cover extent of our primary vegetation baseline changed our predictions. For all of the above we provide all code and links to publicly available data (see 'Data' and 'Software and code' sections)
Randomization	Given we used only secondary ecological data random allocation into experimental groups was not applicable to this study. Measurements in the PREDICTS database are hierarchically structured at a series of nested levels (see 'Research sample' section), which we account for as random intercepts in our mixed-effects models. These random effects account for differences in study design, and to some extent environmental variation predicted by geographical distribution. We also carried out a series of validation analyses indicating that the predictions we draw are likely robust (see 'Reproducibility' section).
Blinding	The analysis in this study used multiple publicly available databases collected prior to this study, meaning blinding was not feasible.

Did the study involve field work? [ ] Yes [x] No

Reporting for specific materials, systems and methods

We require information from authors about some types of materials, experimental systems and methods used in many studies. Here, indicate whether each material, system or method listed is relevant to your study. If you are not sure if a list item applies to your research, read the appropriate section before selecting a response.

Materials & experimental systems

n/a	Involved in the study
☒	☐ Antibodies
☒	☐ Eukaryotic cell lines
☒	☐ Palaeontology and archaeology
☒	☐ Animals and other organisms
☒	☐ Human research participants
☒	☐ Clinical data
☒	☐ Dual use research of concern

Methods

n/a	Involved in the study
☒	☐ ChIP-seq
☒	☐ Flow cytometry
☒	☐ MRI-based neuroimaging
